# Hunger

**Published:** 1871-08

**Authors:** 


### HUNGER.

WHEN the system begins to need nutriment, it sends a fluid from every portion of the body towards the stomach, where it accumulates in little reservoirs, the distension of which causes the sensation of hunger; the fuller they become, the more hungry are we.

This fluid not only gives notice that food is needed, but it has the power of dissolving it, as water dissolves sugar, and thus prepares it for yielding its nutriment to the system. If, therefore, a person eats without an appetite, without being hungry, there being none of this dissolving fluid in the stomach, the food is not dissolved, does not undergo any healthy change; on the contrary, being kept up to the stomach heat of about a hundred degrees, it soon begins to ferment, to decay, to rot; if meat, it literally becomes carrion; if vegetable, it sours; in either case, generating gas and wind, causing unseemly belchings and noisome eructations; or these gases, being confined, distend the stomach, causing pressure against the nerves, originating various pains and discomforts more or less distressing, to last sometimes for hours or half a night, preventing refreshing sleep, to be followed by a day of general discomfort and unfitness for business. Sometimes the stomach becomes so distended with wind that it crowds up against the lungs, preventing them from receiving their proper amount of air, and there follows a distressing feeling of impending suffocation. These same effects follow when too much food is eaten, more than there is fluid in the stomach to dissolve.

